# An Introduction to Longitudinal Synthetic Cohorts for Studying the Life Course Drivers of Health Outcomes and Inequalities in Older Age

**DOI:** 10.1007/s40471-024-00355-1

**Published:** 2024-11-06

**Authors:** Katrina L. Kezios, M. Maria Glymour, Adina Zeki Al Hazzouri

**Affiliations:** 1 Department of Epidemiology, Mailman School of Public Health, Columbia University, New York, NY USA; 2 Department of Epidemiology, Boston University School of Public Health, Boston, MA USA

**Keywords:** Life course research, Synthetic cohort, Longitudinal cohort pooling, Data pooling

## Abstract

**Recent Findings:**

Research on the drivers of health across the life course would ideally be based in diverse longitudinal cohorts that repeatedly collect detailed assessments of risk factors over the full life span. However, few extant data sources in the US possess these ideal features. A “longitudinal synthetic cohort”—a dataset created by stacking or linking multiple individual cohorts spanning different but overlapping periods of the life course—can overcome some of these challenges, leveraging the strengths of each component study. This type of synthetic cohort is especially useful for aging research; it enables description of the long-term natural history of disease and novel investigations of earlier-life factors and mechanisms shaping health outcomes that typically manifest in older age, such as Alzheimer’s disease and related dementias (ADRD).

**Purpose of Review:**

We review current understanding of synthetic cohorts for life course research. We first discuss chief advantages of longitudinal synthetic cohorts, focusing on their utility for aging/ADRD research to concretize the discussion. We then summarize the conditions needed for valid inference in a synthetic cohort, depending on research goals. We end by highlighting key challenges to creating longitudinal synthetic cohorts and conducting life course research within them.

**Summary:**

The idea of combining multiple data sources to investigate research questions that are not feasible to answer using a single cohort is gaining popularity in epidemiology. The use of longitudinal synthetic cohorts in applied research—and especially in ADRD research—has been limited, however, likely due to methodologic complexity. In particular, little guidance and few examples exist for the creation of a longitudinal synthetic cohort for causal research goals. While building synthetic cohorts requires much thought and care, it offers tremendous opportunity to address novel and critical scientific questions that could not be examined in a single study.

## Introduction

Life course research evaluates how timing and duration of exposure to critical structural, environmental, psychosocial, and physiological factors over the entire life span impact disease risk over time [1]. Identifying critical periods of exposure (*e.g.,* birth, childhood, adolescence, early-adulthood, midlife) and/or understanding if and how exposures accumulate across these time periods to increase risk of disease helps inform when and how to intervene [2]. This research is also essential to understanding drivers of health inequalities in older adults. In practice, such research would ideally be conducted using a large and representative longitudinal cohort with lengthy follow-up and repeated, detailed assessments of risk factors and health outcomes at many time points over the complete life span [3–5]. Currently, there is no such cohort available in the US, and only a few international birth cohorts may eventually mature into such data sources [6, 7]. Studies with outcomes ascertained in late life are often initiated in mid-to-late life and rarely include prospective data collected from infancy, childhood or early adulthood—especially not within large and diverse samples [3–5]. At best, retrospective reports or data linkages to administrative sources such as birth records can provide insight into earlier life experiences. However, these sources have important disadvantages. For one, retrospective reports have questionable reliability and may be influenced by later-life health. Additionally, linked data sources typically only provide information at a single, specific age (*e.g.,* birth) offering no opportunity to evaluate whether exposures at different ages are relevant. Even for the foundational task of describing the arc of disease development over the life course, typical age-restricted cohorts are inadequate.

**Fig. 1:**
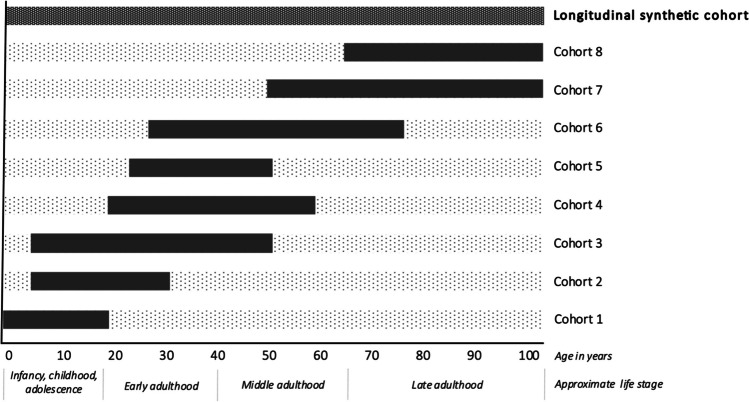
Example schematic for creating a longitudinal synthetic cohort from multiple component cohorts covering different but overlapping periods of the life course. Figure 1 shows the age coverage of data sources contributing to the longitudinal synthetic cohort. Within each cohort, the solid bar indicates the ages/life stages at which data was collected in each cohort, and the dotted bars represent unobserved periods of time. Within the synthetic cohort, the goal is to impute or “fill in” missing information for people—for example, exposure information—*across their life span* from people who are otherwise like them but exist in a different data source in which this information *is* available at a given time point

Epidemiologists are increasingly creating what we call *longitudinal* or *life course synthetic cohorts* (terms we use synonymously) to overcome some of these challenges [3, 4, 8, 9]. We define a longitudinal synthetic cohort as a pooled data set constructed by combining (via stacking or linking) multiple individual cohorts spanning different but overlapping periods of the life course. Figure 1 provides an example illustration of this type of cohort, where the solid bars indicate life course periods when information is available in each cohort and the dotted bars indicate periods when information is unavailable. Within the synthetic cohort, the goal is to impute or “fill in” information across time—for example, exposure information from early life for people who participated in a cohort *without* early life information. This imputation for each person is based on other, similar individuals who are participants in a different data source in which this information *is* available for early life. For example, in Fig. 1, unobserved early life exposure information for individuals in cohorts 4–8 would be imputed or substituted based on information from individuals with observed early life exposure information in cohorts 1–3. The approach to constructing the synthetic cohort may differ depending on the goal of the analysis (discussed more in later sections). In the applied literature, synthetic cohorts are frequently constructed to describe age-related exposure patterns or disease trajectories over longer periods of time than possible in a single cohort [3, 4, 8] (or to achieve this more rapidly than possible in a single cohort, such as in accelerated longitudinal designs [10, 11]). Alternatively, longitudinal synthetic cohorts may be constructed for the purpose of effect estimation, where the goal in the synthetic cohort is to draw valid inference about the life course *effects* of an earlier life exposure on a later life outcome [9]. To answer such questions, the construction of the synthetic cohort is often necessary because no data source exists in which both the exposure and outcome information are measured at the respective time points of interest. Applied examples of synthetic cohorts created for the purpose of effect estimation are less common in the literature than synthetic cohorts used to describe disease development.

While gaining popularity as a method to overcome data limitations in life course research, pooling different longitudinal data sources requires strong assumptions, particularly in the case of effect estimation, to ensure valid findings in the synthetic cohort. However, if constructed carefully, a longitudinal synthetic cohort offers new opportunities to identify and evaluate novel life course determinants and mechanisms of health outcomes occurring in older age [12], such as Alzheimer’s disease and related dementias (ADRD), the causes of which may occur much earlier in the life course than when disease is ultimately diagnosed. In this article, we review current understanding of longitudinal synthetic cohorts for life course research. We begin by discussing chief advantages of longitudinal synthetic cohorts, focusing on their utility for aging/ADRD research to concretize the discussion. We then summarize the conditions needed for valid inference in a synthetic cohort, depending on research goals. We end by highlighting key challenges to creating longitudinal synthetic cohorts and conducting life course research within them.

## Primary Advantages of Longitudinal Synthetic Cohorts

The chief advantage of longitudinal synthetic cohorts is that they offer a solution to a missing data problem. For example, barring linkage to a historical data source, cohorts enrolled at ages 65 + lack exposure/risk factor data prospectively collected in infancy, childhood, adolescence, and middle age. A longitudinal synthetic cohort can be created to fill in this missing risk factor information over time (see Fig. 1), enabling rigorous investigation of age-related exposure patterns or age-specific exposure effects (*e.g.,* identification of critical periods) that may otherwise be impossible or underpowered to detect in any study on its own [3, 4, 8, 12–15]. This could be especially useful in ADRD research, since many risk factors are known to have different associations with ADRD depending on the age when the exposure is assessed. For example, elevated body mass index (BMI) in midlife is associated with higher ADRD risk, but elevated BMI in late-life is often associated with lower (or no) ADRD risk [16, 17]; thus, to understand the life course effects of BMI on ADRD, data across multiple decades of life is essential [16, 18]. Further, with a longitudinal synthetic cohort, risk factors can be assessed starting as early as in utero, allowing researchers to identify how the age and duration of exposure contributes to the incidence, severity, and prognosis of health outcomes later in life. Finally, synthetic cohorts facilitate increases in sample size by pooling data, and these sample size gains allow for examination and characterization of exposures or exposure trajectories that are less common or have smaller effects [12, 14, 19].

Component cohorts making up the longitudinal synthetic cohort may also collect information on the same health outcome but at different points in the life course. Having outcome data filled in over the full life span can provide researchers with deeper understanding about the development of disease over time [20]. For ADRD research, this better equips investigators to answer the question “how early in life does ADRD risk begin?” [5]. That is, if multiple cohorts are combined and, across them, cognition is repeatedly assessed at different ages [21], this could allow for more complete characterization of the course of cognitive impairment and decline preceding a dementia diagnosis. Early manifestations of symptoms consistent with incipient disease can be monitored and the average timing between early symptom onset and eventual ADRD diagnosis can be determined. In some cases, this clearly cannot be accomplished within a single study. For example, the development of Alzheimer’s disease (AD) is thought to span multiple decades [22, 23], yet in vivo imaging of cerebral amyloid burden was first developed in 2004 [24]. Imaging-based descriptions of the development of AD must rely on synthesizing different data sources. In addition to monitoring ADRD development over time, such combined data would also facilitate examination of heterogeneity in cognitive trajectories across different population subgroups [21] and evaluation of life course factors that influence earlier decline [16, 18, 25]. Finally, with large enough samples, synthetic cohort studies of trajectories of rarer forms of ADRD, such as early-onset or familial AD [26], may also be feasible, whereas individual studies often lack sufficient numbers to look at rarer disease subtypes [12, 14, 19, 27].

Longitudinal synthetic cohorts may also be vital resources for distinguishing causal from non-causal findings in the epidemiologic literature. For instance, reverse causation is a potential source of bias for studies of diseases with long prodromal periods, such as dementia. As the disease slowly develops, it may cause subtle changes in physiology or behavior, possibly decades before clinical diagnosis. In ADRD research, reverse causation has been hypothesized as an alternative explanation for the aforementioned lack of association or, in some cases, inverse association between BMI and dementia observed in later life [28]. Early but undetectable manifestations of the disease may cause weight loss, such that *lower* BMI becomes associated with higher risk of dementia. While prior work has attempted to identify the age at which ADRD-related BMI loss begins, results have been conflicting [29, 30]. The magnitude of the reverse causation remains unclear and the possible mechanisms for it are yet to be fully characterized. Longitudinal synthetic cohorts with assessments of cognition across the life span may be promising tools for resolving these issues.

Finally, longitudinal synthetic cohorts also have critical advantages for disparities research and research on ADRD risk within population subgroups not well-represented in any single study. Synthetic cohorts can be much more diverse than any single cohort alone [3, 5, 8, 12, 19, 20]. For example, combining multiple representative datasets enables robust comparison of differences in life course ADRD risk across numerous social categories (*e.g.,* racial and/or ethnic group membership) that might otherwise be excluded from analyses or aggregated into a single category when examined within an individual cohort [31, 32]. In a sufficiently large and diverse synthetic cohort, sample size gains within strata of factors that increase ADRD risk—such as age, gender, educational attainment, and the intersection of these factors—provide power to examine the impact of complex exposure combinations on ADRD risk [5, 33]. Many longitudinal national epidemiologic studies are limited in sample size within strata of single or intersecting social categories, thus the combined synthetic cohort can facilitate much more thorough evaluation of disparities in ADRD risk. Improved understanding of when and how ADRD risk differentially arises and progresses over the life course for different groups, including diverse racial/ethnic groups, rural populations, or socioeconomically disadvantaged groups, is a national priority [26], and synthetic cohorts are promising in advancing this agenda. However, this is not a panacea because the synthetic cohort is constrained by the level of detail in the data originally collected. For example, pooling data may offer new opportunities to include sexual/gender minorities in ADRD research, but only if the component cohorts include data to identify individuals who identify as sexual/gender minorities and experiences relevant to these populations.

## Valid Inference in the Synthetic Cohort

When synthetic cohorts are created for the purposes of estimating the life course effects and mechanisms of an earlier-life exposure on a later-life outcome, it is helpful—if not critical—to use a causal inference framework to guide the synthetic cohort’s construction [2]. Among prior empirical studies that have pooled multiple longitudinal cohorts to create complete life course datasets for the purpose of effect estimation [16, 18, 25, 34–37], limited attention has been paid to causal assumptions (in some studies, considerations are made for confounder adjustment and/or potential sources of selection bias). Yet, effect estimation in synthetic cohorts relies on the same assumptions for causal inference as analyses in individual cohorts, such as no confounding (more formally, exchangeability), well-defined and measured variables (consistency), and positivity (both exposed and unexposed individuals are observed within each covariate stratum), and additionally must satisfy assumptions surrounding data fusion/transportability [38–45].

**Fig. 2:**
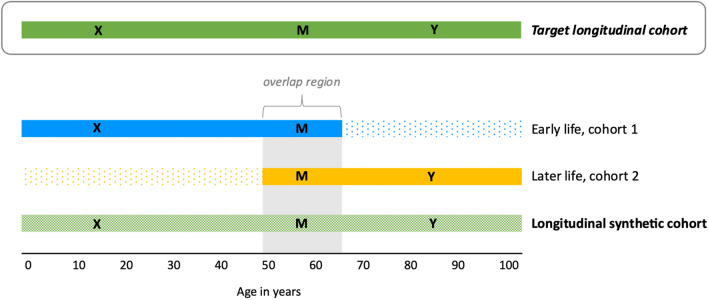
Example longitudinal synthetic cohort pooling schematic. Simple schematic of how cohorts are linked to create a longitudinal synthetic cohort. Here, the aim is to estimate the effect of an early life exposure (“X”) on a later-life outcome (“Y”) in some target population (the target longitudinal cohort) by combining two different but age-overlapping cohorts where information on X is only available in cohort 1 and information on Y is only available in cohort 2. The cohorts may be combined based on shared covariates (collectively represented as “M”), with the aim of allowing the investigator to estimate the target population effect of X on Y in the synthetic cohort in a piecemeal fashion, using partial information from both cohorts. For example, if the target population of interest is cohort 2, then the effect of M on Y is given by cohort 2 and the effect of X on M in cohort 1 is substituted for the unobservable effect of X on M in cohort 2. Thus, in this scenario, valid inference in the synthetic cohort requires the assumption that the effect of X on M in cohort 1 provides an appropriate proxy for the unobservable effect of X on M in cohort 2 (*i.e.,* the quantities are exchangeable)

To help summarize key assumptions needed for valid effect estimation in a longitudinal synthetic cohort, consider the simplified scenario illustrated in Fig. 2 of pooling two cohorts, one younger (cohort 1) and one older (cohort 2)**.** Here, the investigator aims to estimate the effect of an early life exposure (“X”) on a later-life outcome (“Y”) in some target population (the target longitudinal cohort) by combining two different but age-overlapping cohorts where information on X is only available in cohort 1 and information on Y is only available in cohort 2. The cohorts may be combined based on shared covariates measured at similar ages in both cohorts (collectively represented as “M”, and including mediators and confounders of the effect of X on Y). The aim of combining these cohorts based on M is to enable estimation of the target population effect of X on Y in the synthetic cohort in a piecemeal fashion, using partial information from both cohorts (*i.e.,* the effect of X on M from cohort 1 and the effect of M on Y from cohort 2). If we were to assume the target population of interest *was* cohort 2, then a major assumption when combining cohorts 1 and 2 based on shared values of “M” is that the observed relationship between the early life exposure X and merging variables M in cohort 1 is the same as the (unobserved) relationship among these variables in cohort 2. When this condition holds, information on exposure from cohort 1 can potentially be substituted for unobserved exposure information in cohort 2 based on the distributions of M [38, 46]. When this condition is not true (that is, this quantity across cohorts is not exchangeable) invalid causal inference can result in the synthetic cohort. This condition is clearly fulfilled for example if cohorts 1 and 2 are representative samples drawn from the same target population at different ages, but it may be fulfilled in other settings as well. We note that while we present a simple scenario merging just two cohorts, nothing about this approach precludes constructing this type of synthetic cohort from more than two component data sources. For example, one could sequentially link multiple cohorts across time, or they could first stack similarly-aged, concurrent cohorts (to enhance sample size) and then link them with age-overlapping cohorts covering later life periods.

For a concrete example, consider the alignment in time periods and ages of the cohorts shown in Fig. 1, which might be achieved by combining cohorts of Baby Boomers (born ~ 1946–1964). For example, cohort 4 could be the National Longitudinal Survey of Youth 1979 (NLSY79, born 1957–1964) [47] or the Coronary Artery Risk Development in Young Adults study (CARDIA, 1955–1968) [48] while cohort 7 could be the Baby Boomer enrollment cohorts of the Health and Retirement Study (HRS, born 1948–1965) [49] or the Panel Study of Income Dynamics (PSID, born 1950–1968) [14, 50]. Among these example options, the NLSY79 (earlier-life cohort) and HRS and/or PSID (later-life cohorts) may be the best-suited combination since they are all samples from the same target population (US national cohorts) whereas CARDIA represents a different and more select geographic population. Stacking HRS and PSID (similarly-aged, concurrent cohorts) before linking with NLSY79 has the advantage of increasing the sample size of the later-life “outcome” cohort, including within underrepresented subgroups. Despite its different target population, including CARDIA (which has approximately equal numbers of Black and White participants) could be advantageous to increase the number of Black participants in the synthetic cohort. Including a sample drawn from a different target population may be justified on theoretical grounds or empirically by demonstrating observable associations are similar across the cohorts, despite the sampling differences. As in any study, the best combination of datasets largely depends on one’s research purpose.

In applied studies, however, there is often a mismatch between the birth year or other key characteristics of participants in the component cohorts forming the synthetic cohort. Continuing the example above, a mismatch would occur if early-to-midlife information for cohort 3 was provided by The National Longitudinal Study of Adolescent to Adult Health (Add Health, born 1974–1983) [51] and late adulthood information for cohort 8 was provided by The 90 + Study, born in 1913 or earlier [52]. This mismatch would be especially concerning if we suspect that the exposure of interest exhibits period effects. That is, if cohort 3 was composed of individuals who experienced their adolescence in the mid-1980s through early 2000s while cohort 4 were adolescents in the late 1960s through mid-1980s, *and* we expect the relationship between X and M to vary across these different time periods, then the assumption that cohort 3 can serve as an appropriate early life proxy for cohort 4 is likely violated.

In addition to needing the component cohorts to be comparable, for the scenario we have described so far (two cohorts non-overlapping in X and Y are merged on covariates M), successful fusion of the two cohorts to enable causal estimates of the effect of X on Y is only possible when all the pathways linking X and Y are captured within M [9]. If we again think about the goal of creating a synthetic cohort that reflects the X–Y effect in our target population of interest, then the intuition for this condition is that in the synthetic cohort we are trying to capture or reproduce all the ways in which X and Y are associated in the target population. Thus, to capture those associations in the synthetic cohort, we need to combine datasets based on the variables that create them, like confounders and mediators. Linking datasets on mediators helps reproduce the pathways through which X and Y are causally linked. Linking datasets on confounders enables confounder control in the synthetic cohort (*i.e.,* enables us to remove confounding associations in the synthetic cohort); in other words, if we cannot link cohorts by harmonized measures of confounders and then adjust for confounding in the synthetic cohort, we could have residual confounding in the effect estimate in the synthetic cohort. Finally, these special assumptions required for causal inferences based on a synthetic dataset must be considered *on top of* typical threats to validity (*e.g.,* confounding, selection bias, measurement error) within each component cohort. That is, just as in analyses of a single cohort, to produce unbiased estimates of the causal relationship of interest in analyses conducted in the synthetic cohort, sources of bias within each cohort need to be considered and, to the extent possible, prevented and/or corrected for in the data combination process [53].

Finally, as mentioned above, not all longitudinal synthetic cohorts are made for the purposes of causal inference [12, 14, 19]. Synthetic cohorts are sometimes constructed to describe developmental trajectories—for example, changes in cardiovascular disease risk factors [3, 4, 8, 10, 54–57], growth [58], or cognitive function [21] and differences in these trajectories across subgroups [21, 54–56]—over a longer period of time than possible in a single study. Such descriptive studies in synthetic cohorts do not require a “no confounding” assumption because no exposure effect is estimated. However, selection, measurement, and transport considerations are still pertinent in such descriptive synthetic cohorts. Some studies have both descriptive (*e.g.,* modeling life course BMI trajectories in the synthetic cohort) [3] and inferential goals (*e.g.,* estimating the time-specific effects of BMI on dementia across the life course in the synthetic cohort) [16]; in this case, the inferential goal requires all causal assumptions above.

## Key Challenges to Building Unbiased Longitudinal Synthetic Cohorts and Potential Solutions

### Approach for Imputation/Cohort Fusion

There are different ways of approaching the construction of a longitudinal synthetic cohort, especially as it relates to the specific method for filling in missing information across cohorts [14]. In the applied literature, modeling-based approaches are most commonly used, and most often the cohort is constructed for descriptive purposes. For example, in a recent study that conducted a longitudinal integrated data analysis, a pooled data source spanning ages 12 to 105 was created from four component age-overlapping cohorts, and in the harmonized and pooled dataset linear mixed models were used to generate predicted trajectories of cognitive decline (episodic memory and global cognition) from ages 25 to 95 + [21]. Inherent in this approach is the assumption that component cohorts represent samples from the same target population taken at different times over the life course; if this assumption holds, it is reasonable to construct a life course trajectory just using the cohorts with available information over time (as they represent the presumed trajectory for that time period across all cohorts). This approach of using linear mixed models to create life course trajectories in the pooled life course dataset has also been used in other empirical applications [4, 54, 59]. Similar model-based approaches involve imputing (*e.g.,* via multiple imputation with results combined using Rubin’s rules [8]) exposure or risk factor values at unobserved time points prior to construction of trajectories in the pooled cohort via a mixed modeling approach [3, 16, 18, 25].

Little guidance and few examples exist for the creation of a synthetic cohort to support effect estimation. Where applications of longitudinal synthetic cohorts for effect estimation do exist, modeling-based approaches are typically used for their construction [16, 18, 25, 34–37]. However, in such applications, little mention of the decisions underlying the choice of merging variables or other considerations for causal inference are discussed. Importantly, effect estimation requires pooling/merging cohorts based on variables along pathways linking X and Y.. We recently proposed a matching-based approach to combine cohorts for the explicit task of estimating the effect of an early life exposure on an outcome in later-life [9]. In this approach, individuals across cohorts are matched (one-to-many or many-to-many with variance estimates calculated using Rubin’s rules) across cohorts based on having similar values of the merging variables measured at similar ages within the age-overlapping region of the component datasets. Upon matching on M, individuals with outcomes in the later-life cohort are assigned the values of early-life exposure from their earlier-life counterparts [60], and then estimation of the effect of early-life exposure on later-life outcome proceeds among these synthetic individuals comprising the synthetic cohort. This approach is intuitive and is explicit about the assumption that exposure information in the early life cohorts represents the early life unobserved exposure experience for individuals whose data is collected at later time points (an assumption that may be opaquer in approaches which impute or extrapolate/interpolate/smooth over the missing observations across datasets). For this reason, this approach may be preferable if the goal is causal inference (*e.g.,* effect estimation) in the synthetic cohort. However, matching may become inefficient as the number of merging variables increases (where model-based approaches may be more flexible). The specific methods for combining cohorts for different goals (*e.g.,* trajectory modeling, effect estimation), and the appropriate estimands and variance estimators in synthetic cohort analyses corresponding to these different goals, are ongoing areas of methodological development in this literature and a priority area for future research.

### Preventing and Correcting for Bias in the Synthetic Cohort

To ensure (as best possible) that cohorts are exchangeable and transport assumptions are met, researchers must think carefully about the target population of interest and identify component cohorts in which the associations of interest are plausibly reflective of that target – *e.g.,* cohorts that align with the correct sociohistorical period in which they are providing exposure information over the life course. Being clear about the counterfactual of interest can be helpful here. If, for example, the early life cohort is meant to represent the unobserved early life exposure experience of individuals in the later-life/outcome cohort, thinking about the conditions required to make this a reasonable proxy can guide the choice of component data sources. Where this is not achievable and component cohorts reflect samples from different target populations, it is possible that quantitative methods like inverse probability of sampling weighting, outcome model-based approaches like g-computation, or doubly robust methods like targeted maximum likelihood estimation (TMLE) can be used to model and account for differences between the target population and component cohorts [44, 45, 61, 62]. Further, because many population-based cohorts already incorporate sampling weights for representativeness [20], these can be used in the creation of transport weights if the goal is to draw inference to that same target population (*e.g.,* the US population). Additionally, there are ways in which transport/data fusion assumptions can be formally evaluated and tested [63], although the specifics of these approaches extend beyond the scope of this review. Finally, we echo other authors’ perspectives that in the multi-cohort setting, even if transport concerns seem insurmountable, investigating between-study sources of heterogeneity can be an important undertaking in its own right [14, 20]. Leveraging a synthetic cohort to better describe and understand important similarities and differences between its component studies can help advance knowledge of why a given effect exhibits heterogeneity across studies. Such heterogeneity can point researchers toward hypotheses about potential vulnerable subgroups, resiliency factors, etc.

As with transportability, quantitative techniques such as inverse probability of attrition weighting are commonly used to correct for bias due to differential selection processes at the cohort level [53, 64]. One advantage in this multi-cohort setting, is that the individual studies comprising the combined synthetic cohort might be leveraged to help identify, quantify, and correct for selection bias [20]. For example, if each individual study is assumed to be embedded within a larger longitudinal follow-up study, then within each cohort researchers can investigate and model predictors of participation and attrition that also affect disease risk, and estimated probabilities of selection can be used to correct for selection forces within each cohort. After combining individual cohorts, these same selection forces can be investigated and modeled in the complete synthetic dataset to see how sample demographics shift over time due to differential loss to follow-up, participation, and/or survival [9].

### Identifying All Pathways Linking Exposure and Outcome

Needing to identify and account for all pathways linking exposure and outcome may seem like an insurmountable task, but this issue is similar to concerns in the single-cohort setting that we can never know if all confounding pathways are identified and accounted for. Tools developed and promoted in the single-cohort setting, such as bounds and quantitative bias analysis, can be adopted in the synthetic cohort context to evaluate the robustness of findings to the exclusion of important linking pathways in the pooling process [12, 15]. Directed acyclic graphs (DAGs) are also crucial tools for laying out one’s assumptions about the relationships among the variables along these linking pathways, and they may help a researcher choose among several sufficient sets of variables to achieve this pooling task [40]; this could be important considering variable availability and/or measurement concerns when harmonizing and merging on variables across cohorts (detailed below). Related, DAGs could also help identify key downstream variables whose inclusion in M could stand in for many other upstream variables. Again, this could be helpful if both cohorts have measures of these downstream proxy variables but not their upstream determinants. Finally, where the exposure and outcome of interest can change over time (*e.g.,* blood pressure and cognition) and we are interested in determinants of the outcome at the last follow-up period, intermediate measures of these variables can be critical for capturing strong linking pathways from earlier exposure to later outcome (*e.g.,* midlife blood pressure and midlife cognitive function as linking variables for a synthetic cohort analysis of early-adulthood blood pressure and cognitive decline in older age) [9]. However, inclusion of these key variables in M relies on them being repeatedly measured in the component cohorts, including during the period of age-overlap.

Finally, when evaluating the potential contribution of a synthetic cohort, it is important to keep in mind what the overarching purpose of creating the synthetic cohort is. It may be unreasonable to think a synthetic cohort will provide researchers with the exact correct quantity (*e.g.,* point estimate) of interest, given the assumptions required of this approach [12]. Instead, it may be more reasonable to ask, “will the synthetic cohort provide information otherwise unattainable from a single cohort?”. Again, bounds and other tools for sensitivity analyses can be useful here [12, 15]. They may enable a researcher to learn that a positive association is consistently observed between some early life risk factor and later-life outcome in the synthetic cohort. If nothing was known about this relationship without the creation of a synthetic cohort, then there is apparent value gained from the synthetic cohort approach, even in the face of strong assumptions.

### Measurement Issues

Issues of variable measurement and harmonization have been well-described in prior pooling literature, including the context of longitudinal cohort pooling, so we will not repeat these discussions here [4, 12, 14, 15, 19, 20]. We add, however, that in the longitudinal setting we have described, there is the additional complication of needing measures of the same covariates (in “M”) to exist at the matching time period/age [14]. For time-varying covariates, this would mean having measures available at the same or similar age in both cohorts. For time invariant covariates, the variable must simply be collected at some point or wave of the cohort (*e.g.,* baseline). Depending on the time-gap between the exposure of interest (*e.g.,* adolescence) and the outcome of interest (*e.g.,* age 60 cognition), and the time of enrollment for each cohort, it may be that measures of key confounders – especially early life confounders – do not exist in both cohorts. As mentioned in the Introduction, collecting data on these early life factors retrospectively or identifying historical data sources that contain information on early life confounders could offer less-than-perfect solutions. DAGs may again be useful here to identify any upstream variables common across cohorts (*e.g.,* highest parental education) whose control may help reduce or even remove the bias from the early life confounder of interest unmeasured in at least one component cohort (*e.g.,* family income in childhood).

Even if measures of M exist in all component cohorts, to the extent that there is measurement error in the merging variables (before and/or after harmonization), linking paths between X and Y may be insufficiently accounted for, resulting in biased estimates in the synthetic cohort. Thus, comparability of measures in the component cohorts is important for valid inference. However, creating comparable measures across studies via data harmonization is not a straightforward task [19, 20, 27], especially because differences in how questions are asked to study participants may result in different responses. That is, apparently subtle differences in the phrasing of survey questions may influence responses [20]. Psychometric tools and latent variable methods can be helpful for dealing with different measures of the same theoretical construct across a set of studies [20, 59]. An alternative solution is to field an external survey that includes multiple versions of questions about a given risk factor from the different component cohorts (*e.g.,* questions about alcohol use in the NLSY79 vs. the HRS in a recent study) [65] to develop a crosswalk between the different question versions in the different cohorts. This survey sample can act as a “measurement cohort” which allows a direct crosswalk between versions of the question. The crosswalk developed in the measurement cohort can then be applied to the component cohorts to impute each participant’s predicted answer to a different cohort’s question version. Participants across cohorts can then be merged on a particular survey’s version of the measure (which may be an actual response for some and an imputed response for others – see Pederson et al. 2023 for more details [65]). This approach for overcoming measurement issues for data harmonization is a critical and growing area of future research.

## Summary and Conclusions

Here, we review current understanding of longitudinal synthetic cohorts and summarize their chief advantages for conducting life course research. We identify key methodological challenges for unbiased effect estimation in synthetic longitudinal cohorts and discuss how researchers can leverage the creation of the synthetic cohort to identify and correct for these sources of bias. Although the core principle of synthetic cohorts—allowing one group of individuals to represent unobserved values for another group of individuals—may seem like a major assumption, this premise underlies *all* of causal inference.

While creating synthetic cohorts can be challenging and time consuming, harmonization and pooling of existing cohort studies has become a growing area of interest in life course and aging research, and useful resources exist to help researchers identify potential national and international component data sources to pool. These include published Cohort and Data Resource Profiles, as well as University and NIH agency-specific collaborative data resource/consortia web pages, such as the University of Michigan’s Inter-University Consortium for Political and Social Research or the National Institute on Aging’s List of Cohorts and Studies as part of their Common Alzheimer's and Related Dementias Research Ontology (CARDO) initiative [66–68]. We also note that the construction of a life course synthetic cohort for estimating exposure-disease effects (*i.e.,* identifying, harmonizing, and merging by the variables within M) is a research question-specific undertaking; however, code sharing to harmonize certain variables across cohorts may help accelerate the process.

The creation of valid life course data via the combination of multiple existing data sources that cover the entire life span enables researchers to pinpoint when to intervene to prevent or delay the onset of disease. This can be especially important for research on ADRD for which life course datasets including early life exposures and late-life clinically diagnosed disease are lacking. Building synthetic cohorts requires much thought and care, but offers tremendous opportunity to address critical scientific questions in ADRD prevention that would otherwise not be feasible [19].

## Data Availability

No datasets were generated or analysed during the current study.

## References

[1] Jones NL, et al. Life course approaches to the causes of health disparities. Am J Public Health. 2019;109(S1):S48–55. 30699022 10.2105/AJPH.2018.304738 PMC6356123

[2] Kuh D, et al. Life course epidemiology. J Epidemiol Community Health. 2003;57(10):778–83. 14573579 10.1136/jech.57.10.778 PMC1732305

[3] Zeki Al Hazzouri A, et al. Use of a pooled cohort to impute cardiovascular disease risk factors across the adult life course. Int J Epidemiol. 2019;48(3):1004–13. 30535320 10.1093/ije/dyy264 PMC6659365

[4] Hughes RA, Tilling K, Lawlor DA. Combining longitudinal data from different cohorts to examine the life-course trajectory*.* Am J Epidemiol. 2021. 10.1093/aje/kwab190. 10.1093/aje/kwab190 PMC8634562 34215868

[5] Riley A, et al. Why the United States needs a national birth cohort study. Washington: National Academy of Medicine; 2016.

[6] Power C, Kuh D, Morton S. From developmental origins of adult disease to life course research on adult disease and aging: insights from birth cohort studies. Annu Rev Public Health. 2013;34:7–28. 23514315 10.1146/annurev-publhealth-031912-114423

[7] Bynner J. Institutionalization of life course studies. In: Shanahan MJ, Mortimer JT, Kirkpatrick Johnson M, editors. Handbook of the life course: volume II. Cham: Springer International Publishing; 2016. p. 27–58.

[8] Ning H, et al. Development and validation of a large synthetic cohort for the study of cardiovascular health across the life span*.* Am J Epidemiol. 2021. 10.1093/aje/kwab137. 10.1093/aje/kwab137 PMC8633456 33987646

[9] Kezios KL, et al. Overcoming data gaps in life course epidemiology by matching across cohorts. Epidemiology. 2024. 10.1097/EDE.0000000000001761. 10.1097/EDE.0000000000001761 PMC11305898 38967975

[10] Duncan SC, Duncan TE, Hops H. Analysis of longitudinal data within accelerated longitudinal designs. Psychol Methods. 1996;1(3):236–48.

[11] Galbraith S, Bowden J, Mander A. Accelerated longitudinal designs: an overview of modelling, power, costs and handling missing data. Stat Methods Med Res. 2017;26(1):374–98. 25147228 10.1177/0962280214547150 PMC5302089

[12] Zuber S, et al. An integrative approach for the analysis of risk and health across the life course: challenges, innovations, and opportunities for life course research. Discov Soc Sci Health. 2023;3(1):14. 37469576 10.1007/s44155-023-00044-2 PMC10352429

[13] Berry JD, et al. Lifetime Risks of Cardiovascular Disease. N Engl J Med. 2012;366(4):321–9. 22276822 10.1056/NEJMoa1012848 PMC3336876

[14] O’Connor M, et al. Better together: advancing life course research through multi-cohort analytic approaches. Adv Life Course Res. 2022;53:100499. 36652217 10.1016/j.alcr.2022.100499

[15] Downes M, et al. Causal inference in multi-cohort studies using the target trial approach. 2022. arXiv preprint arXiv:2206.11117.

[16] Zeki Al Hazzouri A, et al. Body mass index in early adulthood and dementia in late life: findings from a pooled cohort. Alzheimer's Dement. 2021;n/a(n/a). 10.1002/alz.12367. 10.1002/alz.12367 PMC8809510 33984188

[17] Whitmer RA, et al. Obesity in middle age and future risk of dementia: a 27 year longitudinal population based study. BMJ. 2005;330(7504):1360. 15863436 10.1136/bmj.38446.466238.E0 PMC558283

[18] Yaffe K, et al. Cardiovascular risk factors across the life course and cognitive decline. A pooled cohort study. 2021;96(17):e2212-e2219. 10.1212/WNL.0000000000011747. 10.1212/WNL.0000000000011747 PMC8166431 33731482

[19] Lesko CR, et al. Collaborative, pooled and harmonized study designs for epidemiologic research: challenges and opportunities. Int J Epidemiol. 2018;47(2):654–68. 29438495 10.1093/ije/dyx283 PMC5913631

[20] Curran PJ, Hussong AM. Integrative data analysis: the simultaneous analysis of multiple data sets. Psychol Methods. 2009;14(2):81–100. 19485623 10.1037/a0015914 PMC2777640

[21] Yang YC, et al. An early and unequal decline: life course trajectories of cognitive aging in the United States. J Aging Health. 2023:08982643231184593.22. 10.1177/08982643231184593. 10.1177/08982643231184593 PMC10728348 37335551

[22] Buto PT, et al. Genetic risk score for Alzheimer’s disease predicts brain volume differences in mid and late life in UK biobank participants. Alzheimers Dement. 2024;20(3):1978–87. 38183377 10.1002/alz.13610 PMC10984491

[23] Zimmerman SC, et al. Association of genetic variants linked to late-onset alzheimer disease with cognitive test performance by midlife. JAMA Netw Open. 2022;5(4):e225491. 35377426 10.1001/jamanetworkopen.2022.5491 PMC8980909

[24] Klunk WE, et al. Imaging brain amyloid in Alzheimer’s disease with Pittsburgh Compound-B. Ann Neurol. 2004;55(3):306–19. 14991808 10.1002/ana.20009

[25] Brenowitz WD, et al. Depressive symptoms imputed across the life course are associated with cognitive impairment and cognitive decline. J Alzheimer’s Dis. 2021;Preprint:1–11. 10.3233/JAD-210588 PMC9095065 34420969

[26] National Plan to Address Alzheimer’s Disease: 2020 update. Available from: https://aspe.hhs.gov/index.php/reports/national-plan-address-alzheimers-disease-2020-update. Accessed date 08/31/2021.

[27] Adhikari K, et al. Data harmonization and data pooling from cohort studies: a practical approach for data management. Int J Popul Data Sci. 2021;6(1):1680. 34888420 10.23889/ijpds.v6i1.1680 PMC8631396

[28] Suemoto CK, et al. Body mass index and cognitive function: the potential for reverse causation. Int J Obes (2005). 2015;39(9):1383–9. 10.1038/ijo.2015.83 PMC4758694 25953125

[29] Brenowitz WD. Invited commentary: body mass index and risk of dementia—potential explanations for life-course differences in risk estimates and future research directions. Am J Epidemiol. 2021. 10.1093/aje/kwab095. 10.1093/aje/kwab095 PMC8796800 33831175

[30] Brenowitz WD, et al. Extension of mendelian randomization to identify earliest manifestations of Alzheimer disease: association of genetic risk score for Alzheimer disease with lower body mass index by age 50 years. Am J Epidemiol. 2021;190(10):2163–71. 33843952 10.1093/aje/kwab103 PMC8576370

[31] Kauh TJ, Read JnG, Scheitler AJ. The critical role of racial/ethnic data disaggregation for health equity. Popul Res Policy Rev. 2021;40(1):1–7. 33437108 10.1007/s11113-020-09631-6 PMC7791160

[32] Sue S, Dhindsa MK. Ethnic and racial health disparities research: issues and problems. Health Educ Behav. 2006;33(4):459–69. 16769755 10.1177/1090198106287922

[33] Jaddoe VWV, et al. The LifeCycle Project-EU Child Cohort Network: a federated analysis infrastructure and harmonized data of more than 250,000 children and parents. Eur J Epidemiol. 2020;35(7):709–24. 32705500 10.1007/s10654-020-00662-z PMC7387322

[34] Cohen LP, et al. Association of midlife cardiovascular risk factors with the risk of heart failure subtypes later in life. J Cardiac Fail. 2021;27(4):435–44. 10.1016/j.cardfail.2020.11.008 PMC7987686 33238139

[35] Nair N, et al. Associations of body mass index and waist circumference in young adulthood with later life incident diabetes. J Clin Endocrinol Metab. 2021. 10.1210/clinem/dgab551. 10.1210/clinem/dgab551 PMC8864746 34302728

[36] Zhang Y, et al. Associations of blood pressure and cholesterol levels during young adulthood with later cardiovascular events. J Am Coll Cardiol. 2019;74(3):330–41. 31319915 10.1016/j.jacc.2019.03.529 PMC6764095

[37] Zhang Y, et al. Association between cumulative low-density lipoprotein cholesterol exposure during young adulthood and middle age and risk of cardiovascular events. JAMA Cardiol. 2021;6(12):1406–13. 34550307 10.1001/jamacardio.2021.3508 PMC8459309

[38] Bareinboim E, Pearl J. Causal inference and the data-fusion problem. Proc Natl Acad Sci USA. 2016;113(27):7345–52. 27382148 10.1073/pnas.1510507113 PMC4941504

[39] Breskin A, et al. Fusion designs and estimators for treatment effects. Stat Med. 2021;40(13):3124–37. 33783011 10.1002/sim.8963 PMC8237350

[40] Pearl J, Bareinboim E. Note on “generalizability of study results.” Epidemiology. 2019;30(2):186–8. 30721164 10.1097/EDE.0000000000000939

[41] Pearl J, Bareinboim E. Transportability of causal and statistical relations: a formal approach. In: Twenty-fifth AAAI conference on artificial intelligence. 2011.

[42] Pearl J, Bareinboim E. External validity: from do-calculus to transportability across populations. Stat Sci. 2014;29(4):579–95.

[43] Pearl J, Bareinboim E. Transportability across studies: a formal approach. California Univ Los Angeles Dept of Computer Science; 2011.

[44] Westreich D, et al. Transportability of trial results using inverse odds of sampling weights. Am J Epidemiol. 2017;186(8):1010–4. 28535275 10.1093/aje/kwx164 PMC5860052

[45] Rudolph KE, van der Laan MJ. Robust estimation of encouragement-design intervention effects transported across sites. J R Stat Soc Ser B Stat Methodol. 2017;79(5):1509–25. 10.1111/rssb.12213 PMC5784860 29375249

[46] Cole SR, et al. Illustration of two fusion designs and estimators. Am J Epidemiol. 2022. 10.1093/aje/kwac067. 10.1093/aje/kwac067 PMC10372880 35388406

[47] Rothstein DS, Carr D, Cooksey E. Cohort profile: the national longitudinal survey of youth 1979 (NLSY79). Int J Epidemiol. 2018;48(1):22–22e. 10.1093/ije/dyy133 PMC6380301 29982488

[48] Lloyd-Jones DM, et al. The coronary artery risk development in young adults (CARDIA) study: JACC focus seminar 8/8. J Am Coll Cardiol. 2021;78(3):260–77. 34266580 10.1016/j.jacc.2021.05.022 PMC8285563

[49] Sonnega A, et al. Cohort profile: the Health and Retirement Study (HRS). Int J Epidemiol. 2014;43(2):576–85. 24671021 10.1093/ije/dyu067 PMC3997380

[50] Johnson D, et al. Fifty years of the panel study of income dynamics: past, present, and future. Ann Am Acad Pol Soc Sci. 2018;680(1):9–28. 31666744 10.1177/0002716218809363 PMC6820672

[51] Harris KM, et al. Cohort profile: the national longitudinal study of adolescent to adult health (Add Health). Int J Epidemiol. 2019;48(5):1415–1415k. 31257425 10.1093/ije/dyz115 PMC6857761

[52] Paganini-Hill A, Kawas CH, Corrada MM. Lifestyle factors and dementia in the oldest-old: the 90+ study. Alzheimer Dis Assoc Disord. 2016;30(1):21–6. 25710250 10.1097/WAD.0000000000000087 PMC4561216

[53] Banack HR, et al. Investigating and remediating selection bias in geriatrics research: the selection bias toolkit. J Am Geriatr Soc. 2019;67(9):1970–6. 31211407 10.1111/jgs.16022 PMC9930538

[54] Yang YC, et al. Life-course trajectories of body mass index from adolescence to old age: racial and educational disparities. Proc Natl Acad Sci. 2021;118(17):e2020167118. 33875595 10.1073/pnas.2020167118 PMC8092468

[55] Wills AK, et al. Life course trajectories of systolic blood pressure using longitudinal data from eight UK cohorts. PLoS Med. 2011;8(6):e1000440. 21695075 10.1371/journal.pmed.1000440 PMC3114857

[56] Britton A, et al. Life course trajectories of alcohol consumption in the United Kingdom using longitudinal data from nine cohort studies. BMC Med. 2015;13(1):47. 25858476 10.1186/s12916-015-0273-z PMC4351673

[57] Muniz-Terrera G, et al. Modelling life course blood pressure trajectories using Bayesian adaptive splines. Stat Methods Med Res. 2016;25(6):2767–80. 24770853 10.1177/0962280214532576 PMC5122837

[58] Anderson C, Xiao L, Checkley W. Using data from multiple studies to develop a child growth correlation matrix. Stat Med. 2019;38(19):3540–54. 29700850 10.1002/sim.7696 PMC6767589

[59] Curran PJ, et al. Pooling data from multiple longitudinal studies: the role of item response theory in integrative data analysis. Dev Psychol. 2008;44(2):365–80. 18331129 10.1037/0012-1649.44.2.365 PMC2894156

[60] Andridge RR, Little RJ. A review of hot deck imputation for survey non-response. Int Stat Rev. 2010;78(1):40–64. 21743766 10.1111/j.1751-5823.2010.00103.x PMC3130338

[61] Lesko CR, et al. Generalizing study results: a potential outcomes perspective. Epidemiology (Cambridge, Mass). 2017;28(4):553–61. 28346267 10.1097/EDE.0000000000000664 PMC5466356

[62] Degtiar I, Rose S. A review of generalizability and transportability. Annu Rev Stat Appl. 2023;2023(Volume 10, 2023):501–24.

[63] Luedtke AR, Carone M, van der Laan MJ. An omnibus non-parametric test of equality in distribution for unknown functions. J R Stat Soc Series B Stat Methodol. 2019;81(1):75–99. 31024219 10.1111/rssb.12299 PMC6476331

[64] Weuve J, et al. Accounting for bias due to selective attrition: the example of smoking and cognitive decline. Epidemiology (Cambridge, Mass). 2012;23(1):119–28. 21989136 10.1097/EDE.0b013e318230e861 PMC3237815

[65] Pederson AM, et al. Using an online panel to crosswalk alternative measures of alcohol use as fielded in two national samples*.* medRxiv. 2023. 10.1101/2023.09.13.23295501.

[66] International Alzheimer’s and Related Dementias Research Portfolio. Common Alzheimer's and Related Dementias Research Ontology (CADRO): Category D. Population Studies. 2024. Available from: https://iadrp.nia.nih.gov/about/cadro/Population-Studies-Cohorts-and-Studies. Accessed Date 9/9/2024.

[67] *Cohorts for Life Course Research*. 2024. Available from: https://actri.ucsd.edu/centers-services/portfolio/prsm/cohorts-life-research.html. Accessed Date 9/9/2024.

[68] The Regents of the University of Michigan. *Inter-university Consortium for Political and Social Research (ICPSR)*. 2024. Available from: https://www.icpsr.umich.edu/web/pages/. Accessed date: 9/8/2024.

